# A Viral Nanoparticle with Dual Function as an Anthrax Antitoxin and Vaccine

**DOI:** 10.1371/journal.ppat.0030142

**Published:** 2007-10-05

**Authors:** Darly J Manayani, Diane Thomas, Kelly A Dryden, Vijay Reddy, Marc E Siladi, John M Marlett, G. Jonah A Rainey, Michael E Pique, Heather M Scobie, Mark Yeager, John A. T Young, Marianne Manchester, Anette Schneemann

**Affiliations:** 1 Department of Molecular Biology, The Scripps Research Institute, La Jolla, California , United States of America; 2 Department of Cell Biology, The Scripps Research Institute, La Jolla, California , United States of America; 3 The Salk Institute for Biological Studies, La Jolla, California, United States of America; 4 Division of Cardiovascular Diseases, Scripps Clinic, La Jolla, California, United States of America; Washington University School of Medicine, United States of America

## Abstract

The recent use of Bacillus anthracis as a bioweapon has stimulated the search for novel antitoxins and vaccines that act rapidly and with minimal adverse effects. B. anthracis produces an AB-type toxin composed of the receptor-binding moiety protective antigen (PA) and the enzymatic moieties edema factor and lethal factor. PA is a key target for both antitoxin and vaccine development. We used the icosahedral insect virus Flock House virus as a platform to display 180 copies of the high affinity, PA-binding von Willebrand A domain of the ANTXR2 cellular receptor. The chimeric virus-like particles (VLPs) correctly displayed the receptor von Willebrand A domain on their surface and inhibited lethal toxin action in in vitro and in vivo models of anthrax intoxication. Moreover, VLPs complexed with PA elicited a potent toxin-neutralizing antibody response that protected rats from anthrax lethal toxin challenge after a single immunization without adjuvant. This recombinant VLP platform represents a novel and highly effective, dually-acting reagent for treatment and protection against anthrax.

## Author Summary

Anthrax is caused by the spore-forming, Gram-positive bacterium Bacillus anthracis. The toxic effects of B. anthracis are predominantly due to an AB-type toxin made up of the receptor-binding subunit protective antigen (PA) and two enzymatic subunits called lethal factor and edema factor. Protective immunity to B. anthracis infection is conferred by antibodies against PA, which is the primary component of the current anthrax vaccine. Although the vaccine is safe and effective, it requires multiple injections followed by annual boosters. The development of a well-characterized vaccine that induces immunity after a single injection is an important goal. We developed a reagent that combines the functions of an anthrax antitoxin and vaccine in a single compound. It is based on multivalent display of the anthrax toxin receptor, ANTXR2, on the surface of an insect virus. We demonstrate that the recombinant virus-like particles protect rats from anthrax intoxication and that they induce a potent immune response against lethal toxin when coated with PA. This immune response protected animals against lethal toxin challenge after a single administration without adjuvant. The PA-coated particles have significant advantages as an immunogen compared to monomeric PA and form the basis for development of an improved anthrax vaccine.

## Introduction

Anthrax is caused by the spore-forming, Gram-positive bacterium Bacillus anthracis [1]. The disease is elicited when spores are inhaled, ingested, or transmitted through open wounds in the skin. Inhalational anthrax is the deadliest form of the disease, primarily because it is difficult to diagnose in a timely manner. Disease symptoms are initially nonspecific and systemic dissemination of anthrax toxin can occur prior to antibiotic treatment [2]. The deliberate release of B. anthracis spores in the US in 2001, with the ensuing human fatalities and enormous cleanup costs, has underscored the need for better detection, treatment, and prevention of anthrax.

The toxic effects of anthrax are predominantly due to an AB-type toxin made up of a single receptor-binding B subunit and two enzymatic A subunits [3]. The A subunits are edema factor (EF, 89 kD), an adenylate cyclase that raises intracellular cyclic adenosine monophosphate levels [4], and lethal factor (LF, 90 kD), a zinc protease that cleaves mitogen-activated protein kinase kinases [5,6]. The receptor-binding B subunit is protective antigen (PA), which is initially synthesized as an 83-kD precursor. Upon receptor binding, PA_83_ is cleaved by furin into a 63-kD product that forms heptamers that bind EF to form edema toxin (EdTx) and LF to form lethal toxin (LeTx) [3]. Two anthrax toxin receptors, widely distributed on human cells, have been identified: anthrax toxin receptor/tumor endothelial marker 8 (ANTXR1) [7] and capillary morphogenesis gene 2 (ANTXR2) [8]. Although both receptors bind PA through a 200–amino acid extracellular von Willebrand factor A (VWA) domain, the VWA domain of ANTXR2 has a 1,000-fold higher binding affinity for PA than the VWA domain of ANTXR1. In addition, ANTXR2 has been shown to mediate intoxication in vivo [11]. Recently, the low-density lipoprotein receptor-related protein LRP6 was shown to function as a co-receptor for anthrax toxin internalization, although this finding is controversial [12,13].

The potential use of anthrax as a weapon of bioterrorism has prompted increased efforts to develop better antitoxins and vaccines. Protective immunity to B. anthracis infection is conferred by antibodies against PA, which is the primary component of anthrax-vaccine adsorbed (AVA; Biothrax), the only currently licensed anthrax vaccine in the US. Although AVA is safe and effective, it is molecularly ill-defined, can cause adverse reactions, and is administered in a lengthy immunization schedule (six doses over 18 months) [14]. A second-generation vaccine based on recombinant PA adsorbed on aluminum hydroxide as adjuvant is currently in development. Preliminary data indicate that it is less potent than AVA, and it is likely that several immunizations will be required to confer protection in humans [15]. Thus, the development of a well-characterized vaccine that induces rapid immunity after a single injection remains an important goal.

To develop a reagent that functions both as an anthrax antitoxin and as a molecular scaffold for an efficient anthrax vaccine, we took advantage of an icosahedral virus platform that permits polyvalent display of the extracellular VWA domain of ANTXR2. This platform is based on Flock House virus (FHV), a non-enveloped, icosahedral (*T =* 3) insect virus of the family Nodaviridae [16]. The FHV capsid is composed of 180 subunits of a single type of coat protein, and the icosahedral solid shell encapsidates a bipartite, single-stranded RNA genome. The crystal structure of FHV particles shows that the coat protein contains several surface-exposed loops that can be targeted for insertion of foreign proteins and peptides [17]. Here, we report the synthesis and structural characterization of FHV-VWA_ANTXR2_ chimeric particles and provide evidence for their efficacy as an anthrax toxin inhibitor in vitro and in vivo. In addition, we used the chimeric particles as a scaffold for the multivalent display of PA and show that this complex functions as a potent vaccine against LeTx.

## Results

### FHV-VWA_ANTXR2_ Chimeric Proteins Assemble into Virus-Like-Particles

**Figure 1:**
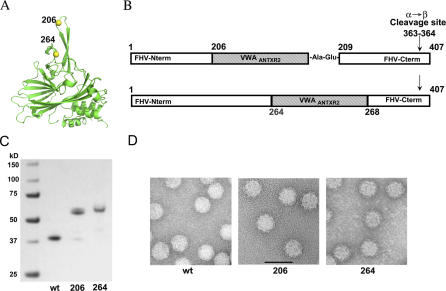
Design and Analysis of FHV-VWA_ANTXR2_ VLPs (A) Ribbon diagram of a single FHV coat protein subunit showing the surface-exposed loops into which the VWA domain of ANTXR2 was inserted. (B) Schematic diagram showing structure of FHV-VWA_ANTXR2_ chimeric proteins 206 (top) and 264 (bottom). Numbers refer to amino acid positions in the FHV coat protein. The assembly-dependent autocatalytic maturation cleavage occurs between Asn 363 and Ala 364. (C) Electrophoretic analysis of purified VLPs on a 10% Bis-Tris gel stained with Simply Blue (Invitrogen). Lane 1, molecular weight markers; lane 2, wt FHV VLPs; lane 3, FHV-VWA_ANTXR2_ chimera 206; lane 4, FHV-VWA_ANTXR2_ chimera 264. (D) Electron micrographs of gradient-purified wt and chimeric VLPs negatively stained with uranyl acetate. Bar = 500 Å.

The VWA domain of ANTXR2 forms a compact structure that adopts a Rossmann-like α/β-fold with a metal ion-dependent adhesion site motif that is involved in PA binding [9,18]. The N and C termini of this domain, residues C39 and C218, respectively, are closely juxtaposed, thereby permitting, in principle, genetic insertion into a loop on a carrier protein. Modeling studies of the FHV coat protein subunit indicated that two surface-exposed loops at amino acid positions 206 and 264 would accommodate the 181 amino acid ANTXR2 VWA domain without disrupting coat protein assembly into virus-like particles (VLPs) (Figure 1A). Based on these predictions, two chimeric proteins were generated. In FHV-VWA_ANTXR2_ chimera 206, the VWA domain and a C-terminal two–amino acid linker (Ala-Glu) replaced FHV coat protein residues 207–208 (Figure 1B). In FHV-VWA_ANTXR2_ chimera 264, the VWA domain replaced FHV residues 265–267. The chimeric proteins were expressed in Sf21 insect cells using recombinant baculovirus vectors. In this system, wild-type (wt) FHV coat protein forms VLPs whose high resolution structure is virtually indistinguishable from that of native virions (unpublished data). However, VLPs contain random cellular RNA instead of the FHV genome and are therefore not infectious [19].

Putative chimeric VLPs were purified from the cells by sucrose gradient centrifugation and material sedimenting at a position similar to that observed for native virions harvested and analyzed by SDS-PAGE. As shown in Figure 1C, both samples contained a major protein and a slower migrating minor protein of the appropriate molecular weights (≈63 kD, the combined molecular weight of the 43-kD FHV coat protein and the 20-kD ANTXR2 VWA domain). Since the FHV coat protein undergoes a spontaneous cleavage reaction after assembly of particles (Figure 1B) [20], the minor protein likely represented the unprocessed precursor protein, whereas the major protein represented the post-assembly cleavage product. Capsid proteins representing chimera 264 migrated more slowly through the gel than those representing chimera 206, even though the amino acid composition of the two polypeptides was virtually identical. The reason for this differential behavior is not known but could reflect subtle differences in denaturation of the proteins under SDS-PAGE conditions.

Electron microscopy of negatively stained samples confirmed the presence of VLPs in the gradient-purified material (Figure 1D). Compared to the smooth exterior of native FHV virions, the surface of the chimeric particles was rough and distinct protrusions were visible. The appearance of the particles suggested that they were filled with RNA, as stain did not penetrate the interior. This conclusion was supported by the sedimentation rate of the VLPs, which was indistinguishable from that of wt FHV (not shown).

### FHV-VWA_ANTXR2_ VLPs Protect Cultured Cells from Intoxication

**Figure 2:**
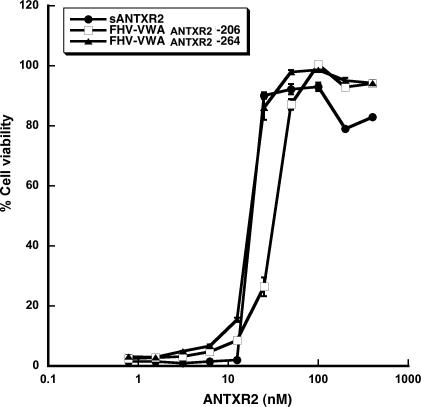
Dose Response Curve Showing Cell Viability as a Function of Antitoxin Concentration CHO-K1 cells were incubated with PA-LF_N_DTA in the presence of increasing concentrations of sANTXR2, chimera 206, or chimera 264. After incubation at 37 °C for 48 h, cell viability was determined with Celltiter-glo (Promega). Data points represent the mean ± SD values for triplicate samples. All data were normalized to untreated cell controls.

The soluble, monomeric ANTXR2 VWA domain (sANTXR2), expressed and purified from mammalian cells, was previously shown to effectively block entry of LeTx into susceptible cells by competing with cellular ANTXR2 for binding to PA [10]. PA has a very high binding affinity for sANTXR2 (K_d_ = 170 pM) and dissociates extremely slowly from this receptor decoy (the half-life of the complex is approximately 17 h) [21]. We used the same approach to test the inhibitory activity of FHV-VWA_ANTXR2_ VLPs. Namely, the assay employed CHO-K1 cells and a modified form of LeTx, PA/LF_N_-DTA, in which the N-terminal portion of LF was fused to the catalytic portion of diphtheria toxin A-chain [22]. This recombinant toxin efficiently kills CHO-K1 cells within 48 h and uses the same PA-dependent entry mechanism as wt LF. The assay revealed that chimera 264 protected cells as efficiently as sANTXR2, whereas a higher concentration of chimera 206 was required to achieve protection of the cells (Figure 2). The corresponding IC_50_ values for sANTXR2 and chimera 264 were 19.70 ± 0.87 nM and 18.50 ± 0.36 nM, respectively, while the IC_50_ was 32.71 ± 0.61 nM for chimera 206. Thus, chimera 264 performed as well as the highly potent, monomeric sANTXR2 inhibitor in this assay. To confirm the ability of the particles to neutralize native LeTx, a macrophage-based toxin neutralization assay was performed with chimera 264. The assay revealed that the particles protected RAW264.7 cells efficiently from a mixture of PA and wt LF, and the measured IC_50_ was 39.8 ± 2.2 nM.

### FHV-VWA_ANTXR2_ Chimera 264 Protects Rats from LeTx Challenge

We next tested whether the chimeric particles were capable of protecting rats against LeTx challenge as was demonstrated previously for sANTXR2 [10]. In vivo experiments were only performed with chimera 264 because it had shown higher potency in the cell intoxication assay (Figure 2). As a positive control, sANTXR2 was used in parallel. Male Fisher 344 rats were inoculated intravenously with 5 minimal lethal doses (MLDs) of LeTx either in the presence or absence of chimera 264 or sANTXR2 as previously described [10]. As shown in Table 1, both chimera 264 and sANTXR2 completely protected the animals when used at a molar ratio of 2:1 (ANTXR2:PA). Moreover, the animals did not exhibit any symptoms of intoxication such as agitation, respiratory distress, or hypoxia. Injection vehicle (PBS) or wt FHV, used as a negative control, had no protective effect. While neither chimera 264 nor sANTXR2 were able to protect rats when used at a 10-fold lower concentration (molar ratio of ANTXR2:PA = 0.2:1), they each caused a delay in the time to death compared to the LeTx control (Table 1). The delay was notably longer for chimera 264 (89 min) than sANTXR2 (77 min), and the difference was highly significant (*p* = 0.0046), suggesting increased therapeutic potency of the multivalent particles over monomeric sANTXR2 as an inhibitor of the anthrax toxin. It will be interesting to determine whether different pharmacokinetic profiles are observed in vivo for sANTXR2 and chimera 264.

**Table 1:**
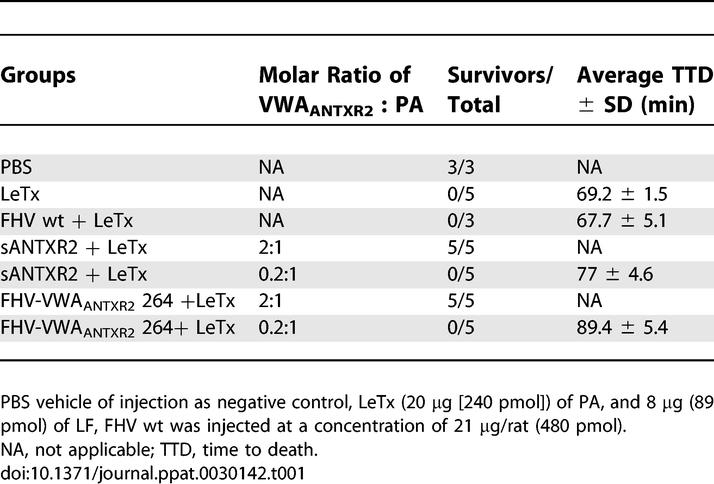
In Vivo Intoxication Experiments in Fisher 344 Rats

### Structural Analysis of FHV-VWA_ANTXR2_ VLPs

**Figure 3:**
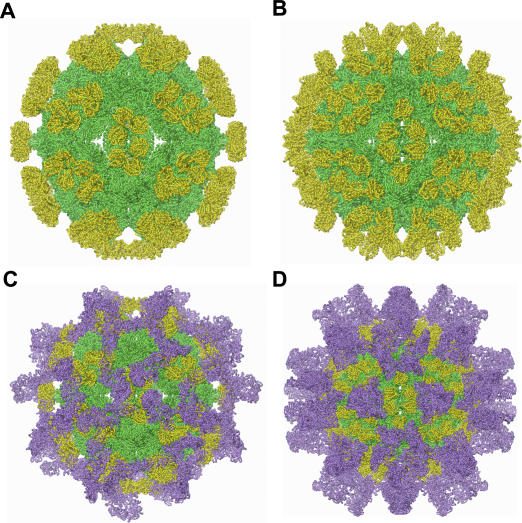
Three-Dimensional Models of FHV-VWA_ANTXR2_ VLPs with and without Bound PA_83_ (A, B) Pseudoatomic models of FHV-VWA_ANTXR2_ chimeras. X-ray coordinates of FHV capsid protein (green) and ANTXR2 VWA domain (yellow) were docked into the cryoEM density of chimera 206 (A) and chimera 264 (B). Panels show surface views of the particles in the absence of the cryoEM density maps. Note the different distributions of the ANTXR2 domains on the surfaces of the VLPs. (C, D) In silico model of PA_83_ bound to the surface of FHV-VWA_ANTXR2_ chimeras. PA_83_ (purple) was modeled onto the surface of chimera 206 (C) and chimera 264 (D) using the known high resolution X-ray structure of the ANTXR2-VWA/PA_63_ complex as a guide [9]. Panels show surface views of the entire particles to illustrate the difference in occupancy of PA_83_ on the two chimeras.

Electron cryomicroscopy and image reconstruction of the FHV-VWA_ANTXR2_ VLPs showed that, compared to wt FHV particles (Figure S1C), both chimeric particles displayed additional density at higher radius (Figure S1A and S1B), which is in agreement with the protrusions that were visible in negatively stained samples (Figure 1D). To define the arrangement of the VWA domains on the surface of the chimeric particles, pseudoatomic models were generated by fitting the X-ray coordinates of the FHV coat protein subunit and the ANTXR2 VWA domain into the cryoEM density maps (Figures 3A, 3B, S2A, and S2B). The models revealed that in chimera 206 the VWA domains were closely juxtaposed at the quasi 3-fold axes. Two of the three VWA domains in each asymmetric unit closely interacted with their 2-fold related counterparts, thereby creating an offset cluster of six domains. In contrast, the insertion site chosen for chimera 264 allowed for wider spacing and more even distribution of the individual VWA domains on the particle surface.

To investigate the accessibility of PA to the VWA domains, PA_83_ was computationally docked onto the VWA domains of the pseudoatomic models of chimeras 264 and 206 using the X-ray structure of PA complexed with the ANTXR2 VWA domain as a guide [9,18]. It was evident that chimera 264 could accommodate significantly more PA molecules than chimera 206 given the wider spacing of the VWA domains on this particle (Figures 3C, 3D, S3A, and S3B). Specifically, each subunit at the 5-fold axes and three of the six subunits around the quasi-6-fold axes could bind PA_83_ without steric interference, giving a total occupancy of 120 PA molecules per particle. In contrast, due to the close juxtaposition of the VWA domains on chimera 206, a maximum occupancy of 60 PA molecules per particle was predicted. These predictions were in close agreement with results from biochemical analyses of complexes formed between the particles and PA_83_ under saturating conditions. Specifically, gel electrophoresis combined with densitometric analysis showed that chimera 206 could bind an average of 90 PA_83_ ligands, whereas chimera 264 bound an average of 130 PA_83_ ligands (Figures S4A and 4B). Together, these results were consistent with the observation that a higher concentration of chimera 206 was required to protect cells from intoxication with PA/LF_N_-DTA (Figure 2).

### FHV-VWA_ANTXR2_ Particles Serve as a Highly Effective Vaccine Platform

The observation that FHV-VWA_ANTXR2_ chimera 264 functioned as a binding surface for multiple copies of PA suggested that a complex of the two components might constitute an effective antigen for induction of PA-specific antibodies. To test this, complexes were prepared by mixing chimera 264 with an excess of PA_83_, and unbound PA_83_ was removed by ultracentrifugation. Electron microscopic analysis showed that PA_83_ formed thin protrusions emanating from the capsid surface (not shown).

**Figure 4:**
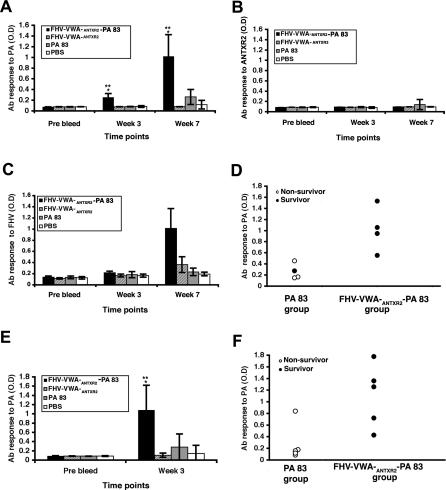
Antibody and LeTx Challenge Response of immunized Rats (A–D) Rats (four per group) were immunized with FHV-VWA_ANTXR2_–PA_83_ complex, FHV-VWA_ANTXR2_ chimera alone, PA_83_, or PBS and boosted 4 wk later. Serum samples were collected prior to as well as 3 and 7 wk after immunization and tested by ELISA for IgG-specific antibody response to (A) PA, (B)VWA_ANTXR2_, and (C) FHV coat protein. Data represent the mean ± SD of animals in the respective groups and are shown for the 1:1,000 serum dilution in panels (A) and (B) and for the 1:100 serum dilution in panel (C). In panel (A) at week 3, * indicates *p* = 0.003 compared to PBS control and ** indicates *p* = 0.003 compared to PA_83_ alone. At week 7, * indicates *p* = 0.005 compared to PBS control and ** indicates *p* = 0.012 compared to PA_83_ alone. (D) Relationship between anti-PA antibody level and survival of individual rats following challenge with 10 MLDs of LeTx. (E, F) Rats (five per group) were immunized once with FHV-VWA_ANTXR2_–PA_83_ complex, FHV-VWA_ANTXR2_ chimera alone, PA_83_, or PBS. Serum samples were collected prior to and 3 wk after immunization, diluted 1:100, and tested for IgG-specific antibody response to PA (E). Data represent the mean ± SD of animals in the repective groups. At week 3, * indicates *p* < 0.0001 compared to PBS control and ** indicates *p* = 0.001 compared to PA_83_ alone. (F) Relationship between anti-PA antibody level and survival of individual rats following challenge with 10 MLDs of LeTx.

For immunogenicity studies, rats (four per group) received two subcutaneous injections (0 and 3 wk) of either 2.5 μg of PA_83_, 5.4 μg of particle–PA complex (molar equivalent of 2.5 μg of PA_83_ assuming an occupancy of 120 VWA domains), or 2.9 μg of chimera 264. No adjuvants were employed in these experiments. The ELISA assay of pre- and post-inoculation sera showed that animals immunized with the complex had significantly higher levels of anti-PA antibody than animals receiving PA alone both at week 3 (*p* = 0.0028 compared to PA alone) and after boosting (week 7; *p* = 0.0118 compared to PA alone) (Figure 4A). No significant antibody response against ANTXR2 VWA was detected in these animals, but a response against FHV protein was observed after the boost in rats that were immunized with the complex (Figure 4B and 4C). Why animals immunized with chimera 264 alone did not mount a similar immune response against FHV protein is not clear. The presence of PA may somehow enhance the response to FHV or may influence the localization or interaction of FHV particles with antigen-presenting cells, causing a difference in the observed anti-FHV antibody response.

To test whether the anti-PA antibodies were protective against anthrax toxin, the rats were challenged by intravenous inoculation with 10 MLDs of LeTx. All animals that had been immunized with the particle–PA complex survived, whereas all but one of the animals in the PA_83_ group died (Figure 4D). Survival versus death correlated well with the level of anti-PA antibody detected in the sera. However, the animal in the PA-only group that survived LeTx challenge had a serologic response to PA that was well below that of a non-surviving animal in the same group (Figure 4D).

Based on the observation that animals immunized with the particle–PA complex showed a significant level of anti-PA antibody as early as 3 wk after the first immunization (Figure 4A), we investigated whether animals could be protected against LeTx after a single injection. Rats (five per group) were immunized once with increased doses of the particle–PA complex (10.8 μg), PA_83_ (5 μg), or chimera 264 (5.8 μg) as a control. After 3 wk, the animals were bled and challenged 1 wk later with 10 MLDs of LeTx. All rats that were immunized with the particle–PA complex survived, whereas all other animals died (Figure 4F). Of those that died, one animal had a serologic antibody response to PA that was greater than that of two animals in the group of survivors (Figure 4E and 4F), indicating that the magnitude of the antibody response is not a reliable predictor of protection. Taken together, our results show that multivalent display of PA on the FHV-VWA_ANTXR2_ scaffold yields a significant advantage over monovalent, soluble PA as an immunogen for anthrax toxin.

## Discussion

In this study we have developed a novel reagent that combines the functions of anthrax antitoxin and vaccine in a single compound. It is based on multivalent display of the ANTXR2 VWA domain on the surface of the icosahedral insect nodavirus FHV. We demonstrate that the recombinant VLPs protect cultured cells and rats from anthrax intoxication as efficiently as the highly potent sANTXR2 receptor decoy and that they induce a potent immune response against LeTx when coated with PA. This immune response was neutralizing in vitro and protected animals against LeTx challenge following a single administration without adjuvant.

The motivation for immunogenicity studies was based on the assumption that polyvalent display of PA would induce a more potent immune response than monomeric, recombinant PA, which is currently being developed as a second-generation anthrax vaccine [15,23]. Ordered arrays of antigens are known to permit particularly efficient cross-linking of B cell receptors, which in turn leads to faster and more robust B cell proliferation [24–26]. Given the exceptionally tight binding of PA to ANTXR2 under natural conditions (K_d_ = 170 pM) [21], we reasoned that complexes formed between chimera 264 and PA would be sufficiently stable to serve as an immunogen in vivo*.* In support of this notion, results from in vitro cell intoxication experiments indicated that the complexes were stable for at least 40 h at 37 °C. Based upon a recent observation that naturally occurring PA neutralizing antibodies do not bind to the receptor-binding surface of PA [27], we reasoned that PA immobilized on these particles should be able to elicit a protective immune response. Indeed, rats survived LeTx challenge 4 wk after a single injection of the VLP-PA complex, whereas animals injected with an equivalent amount of recombinant PA died. This result suggested rapid production of neutralizing antibodies in the absence of adjuvant, two key goals for the development of third-generation anthrax vaccines. No significant antibody response to ANTXR2 was observed, presumably because there are only two–amino acid differences between human ANTXR2 displayed on the particle and endogenous rat ANTXR2 [11].

An essential next step will be to characterize the neutralizing antibody response in individual animals after primary and secondary immunization. An important component of this analysis will be to determine the mechanism by which toxin neutralization occurs. For example, we noticed a slight difference in antibody response after primary and secondary immunization and a wide range of antibody titers between individual animals (Figure 4). It will be of key interest to establish whether these differences correlate with epitope specificity or are based on other immunologic parameters. In addition, it will be critical to confirm our findings in a B. anthracis spore challenge model, and studies to this end are currently underway.

Because the chimeric particles are expressed from an mRNA that contains only the coding sequence of the modified FHV coat protein while all other FHV sequences are missing, the resulting VLPs are not infectious and thus cannot replicate in mammalian tissues [19]. Even native FHV particles are unable to initiate infection in mammals, as they do not carry the FHV receptor, and because FHV cannot replicate at temperatures above 31 °C [28]. We have also demonstrated previously that FHV VLPs expressed from baculovirus vectors in Sf21 cells do not contain baculoviral or cellular DNA [19], thus ruling out potential integration of foreign DNA into mammalian genomes. Based on these properties, the chimeric particles can be expected to have a desirable safety profile for applications in animals and humans.

The idea of combining the functions of anthrax vaccine and antitoxin in a single reagent has been explored previously. Aulinger et al. [29] demonstrated that a dominant-negative, inhibitory form of PA, DNI-PA, can elicit an antibody response that protects mice from LeTx challenge. DNI-PA forms mixed heptamers with wt PA and thereby acts as an antitoxin to block toxin translocation both in vitro and in vivo [30]. However, even after two injections in the presence of adjuvant there was only a weak antibody response to DNI-PA, and a third injection had to be performed to generate a sufficient antibody response to protect against LeTx challenge [29].

While the potency of the nanoparticles as a vaccine is most likely due to polyvalent display of PA, polyvalency is less of a factor in the function of the particles as an antitoxin given the extremely high affinity between PA and ANTXR2. Moreover, since PA binds as a monomer to the particles, little, if any, polyvalent effect is to be expected. In fact, we detected no significant difference in IC_50_ when comparing nanoparticles with soluble ANTXR2 in cell intoxication assays. That polyvalency increases the affinity between a ligand and its target receptor is a well-established phenomenon [31]. Recently, Rai et al. [32] reported that “pattern matching” is an important parameter for polyvalency to reach its maximum potential. With this approach, they achieved similar IC_50_ values in cell intoxication assays for liposomes containing inhibitory peptides that block LF binding to the PA heptamer as we observed for our nanoparticles. However, the functionalized liposomes described in their study are without a vaccine application.

In vivo potency of viral nanoparticles is also significantly determined by their pharmacokinetic parameters. Such parameters have recently been reported for viral nanoparticles derived from the plant virus cowpea mosaic virus [33]. It will be important to determine whether there are significant differences in the plasma clearance kinetics and biodistribution of soluble ANTXR2 versus ANTXR2-containing nanoparticles.

The VWA domain of ANTXR2 was a particularly appealing candidate for insertion into a loop of the FHV coat protein because the N and C termini are only separated by 4.8 Å in the native structure [34]. In addition, this domain adopts a compact Rossmann-like α/β-fold that can evidently form independently within the context of a larger protein while not interfering with accurate folding of the carrier protein. This hypothesis was supported by the observation that the high-resolution structure of the VWA domain could be fitted easily into the cryoEM density maps. To our knowledge, hepatitis B virus is the only other virus for which icosahedral surface display of an entire protein in its biologically active conformation has been demonstrated. In that case, genetic insertion of the green fluorescent protein in a surface-exposed loop of the core protein resulted in efficient formation of fluorescent hepatitis B virus capsids [35].

In principle, it should be possible to expand the use of the FHV platform to display additional anthrax antigens either in the presence or absence of the ANTXR2 VWA domain. Specifically, direct insertion of peptides or entire domains derived from PA, LF, and EF may be feasible as long as the termini of the domains are in close enough proximity for insertion into the FHV coat protein loops. It is also conceivable that the two insertion sites at positions 206 and 264 could be used in combination to create particles with multiple functionalities. This could greatly enhance the protection afforded by the resulting particles.

Numerous other strategies are being pursued to develop improved anthrax vaccines, including PA-expressing *Salmonella* [36] and B. subtilis [37], adenovirus encoding PA domain 4 [38], rabies virus encoding GP-PA fusion protein [39], and bacteriophage T4 particles decorated with PA-hoc fusion proteins [40–42]. None of these, however, combine the function of vaccine and antitoxin. In those cases where immunized animals were challenged with LeTx or anthrax spores, only the adenovirus construct provided complete protection after a single immunization [38]. The strategy most comparable to that described in our study involves non-covalent surface display of intact proteins and protein complexes on bacteriophage T4 particles. The prolate lattice of the T4 capsid permits efficient surface presentation of anthrax toxin through in vitro addition of Hoc- and/or Soc protein fusions with PA, LF, or EF to *hoc^−^soc^−^* phage either separately or in combination [40,42]. Mice immunized with phage displaying PA, EF, and LF generated high levels of neutralizing antibodies [41], but results from toxin or spore challenge experiments have not yet been reported.

In summary, we have developed a reagent that serves a dual purpose in combating B. anthracis infection. It functions as a competitive inhibitor of anthrax toxin in vivo, suggesting that it could be useful as a therapeutic compound, particularly in combination with standard antibiotic therapy. In addition, when complexed with PA, it has significant advantages as an immunogen compared to monomeric PA and thus forms the basis for development of an improved anthrax vaccine.

## Materials and Methods

### Construction of recombinant baculoviruses.

DNA fragments encoding FHV coat protein-ANTXR2 VWA domain chimeras were generated by overlap extension PCR using *Pfu* polymerase [43]. Three DNA fragments containing the nucleotide sequence for the N-terminal portion of the coat protein, the ANTXR2 VWA domain (GenBank accession number AY23345, nts 115–657, amino acids 38–218), and the C-terminal portion of the coat protein, were initially generated. The template used for generating segments containing the FHV coat protein sequence was plasmid pBacPAK9RNA2δ [44], which contains the full-length cDNA of FHV RNA2 in baculovirus transfer vector pBacPAK9 (BD Biosciences). The template used for generating the segment containing the ANTXR2 VWA coding sequence was a derivative of plasmid PEGFP-N1 [8]. Following overlap extension PCR, the full-length product was digested with BamH1 and XbaI, gel-purified, and ligated into equally digested pBacPAK9. The transfer vector was amplified in E. coli strain DH5α, purified, and sequenced to confirm the presence of the ANTXR2 VWA sequence and to ensure the absence of inadvertent mutations. Recombinant baculoviruses AcFHV-VWA_ANTXR2_-264 and AcFHV-VWA_ANTXR2_-206 were generated by transfecting Sf21 cells with a mixture of transfer vector and linearized Bsu36I-linearized BacPAK6 (BD Biosciences) baculovirus DNA as described previously [43].

### Cells and infection.

Spodoptera frugiperda cells (line IPLB-Sf21) were propagated and infected as described previously [19]. Trichoplusia ni cells were propagated and infected as described by Dong et al. [45] except that EX-CELL 401 medium was replaced with ESF921 (Expression Systems).

### Purification of virus-like particles (VLPs).

VLPs were purified from *T.ni* suspension cultures 5 to 6 days after infection. NP-40 substitute (Fluka) was added to the culture to a final concentration of 1% (v/v) followed by incubation on ice for 10–15 min. Cell debris was pelleted by centrifugation in a Beckman JA-14 rotor at 15,300*g* for 10 min at 4 °C. VLPs in the supernatant were precipitated by addition of NaCl to a final concentration of 0.2 M and polyethylene glycol 8000 (Fluka) to a final concentration of 8% (w/v) and stirring the mixture at 4 °C for 1 h. The precipitate was collected by centrifugation at 9,632*g* for 10 min at 4 °C in a JA-14 rotor and resuspended in 50 mM Hepes buffer (pH 7.5). Insoluble material was removed by centrifugation at 15,300*g* for 20 min at 4 °C. VLPs in the clarified supernatant were pelleted through a 4-ml 30% (w/w) sucrose cushion in 50 mM Hepes (pH 7.5) by centrifugation in a Beckman 50.2 Ti rotor at 184,048*g* for 2.5 h at 11 °C. The pellet was resuspended in 50 mM Hepes buffer (pH 7.5) and loaded onto a 10%–40% (w/w) sucrose gradient in the same buffer. The gradients were spun in a Beckman SW 28 rotor for 3 h at 103,745*g*. VLPs were collected from the gradient by inserting a needle below the VLP band and aspirating the material into a syringe. Alternatively, gradients were fractionated with continuous absorbance at 254 nm on an ISCO gradient fractionator at 0.75 ml/min and 0.5 min per fraction. Fractions containing VLPs were then dialyzed against 50 mM Hepes, (pH 7.5) and concentrated to 1–5 mg/ml using a centrifugal concentrator with a 100,000 MW cut off (Amicon, Millipore). The final protein concentration was determined by BCA assay (Pierce Chemicals) and purity was evaluated by densitometry (FluorChem SP, Alpha Innotech) after electrophoresis on a 10% Bis-Tris gel stained with Simply Blue (Invitrogen).

### Electron microscopy.

Samples of gradient-purified VLPs were negatively stained with 1% (w/v) uranyl acetate. A drop of each sample was adsorbed to a glow-discharged, collodion-covered copper grid and allowed to adsorb for 1–2 min. Excess solution was removed by blotting with filter paper. The grids were washed and blotted with filter paper three times by floating on droplets of 50 mM Hepes (pH 7.5). Each grid was then treated three times with a drop of 1% uranyl acetate solution and left in the third drop for 1–2 min prior to blotting and air drying. The samples were viewed in a Philips/FEI CM 100 transmission electron microscope at 100 kV.

### Electron cryomicroscopy and image reconstruction.

Frozen-hydrated samples were prepared using standard methods [46]. In brief, an aliquot of the sample was applied to a glow-discharged Quantifoil holey carbon-coated grid (2/4 Cu-Rh), blotted with filter paper, and rapidly plunged into liquid ethane. Low-dose electron micrographs of FHV-VWA_ANTXR2_-264 VLPs were recorded onto Kodak SO163 film at a magnification of 45,000× on a Philips/FEI CM120 transmission electron microscope. For FHV-VWA_ANTXR2_-206 VLPs, low-dose micrographs were recorded on a CCD camera at a magnification of 50,000× on a Philips/FEI Tecnai20 transmission electron microscope. The grids were maintained at −180 °C using a Gatan 626 cryo-stage. Micrographs with minimal astigmatism and drift, as assessed by visual inspection and optical diffraction, were digitized with a Zeiss microdensitometer (Z/I Imaging), giving a step-size of 3.1 Å on the specimen. Images recorded on the CCD camera had a step-size of 2.26 Å. Particle images were extracted with the program X3D [47] and were processed by polar Fourier transform methods using the program PFT [48]. A previously calculated model of wt FHV [49] was used as the starting model. Initial refinement cycles were restricted to the radii spanning the FHV capsid and then relaxed to incorporate the extra domains. Using a Fourier shell correlation cut-off value of 0.5, the FHV-VWA_ANTXR2_-206 and FHV-VWA_ANTXR2_-264 maps were refined to resolutions of 25 and 23 Å, respectively.

### Generation of pseudoatomic models.

The atomic coordinates of the FHV coat protein subunit and the VWA domain of ANTXR2 (PDB ID: 1SHT) were used to generate a pseudoatomic model of the FHV-VWA_ANTXR2_-206/264 VLPs. Specifically, the models were created with the program O [50] by visually positioning the ANTXR2 VWA domains at the surface of the FHV structure and adjusting for overlap. The models were then further refined against the structure factor amplitudes derived from the cryoEM density using the program CNS [51]. Individual subunits and domains of the FHV-VWA_ANTXR2_ chimera were allowed to move independently as rigid bodies and subjected to five rounds of 20 cycles of rigid body refinement. PA_83_ molecules were docked onto the resulting FHV-VWA_ANTXR2_-206/264 models using the structure of PA_63_ complexed with the ANTXR2 VWA domain (PDB ID: 1T6B) as a guide [9]. Once all 180 ANTXR2 VWA domains on the FHV-VWA_ANTXR2_ chimera were populated with PA_83_ molecules, a minimal number of PA molecules were selectively removed to relieve steric clashes with neighboring PA molecules.

### Quantification of PA_83_ bound to chimeras 206 and 264.

Recombinant PA_83_ (List Biological Laboratories) in 5 mM Hepes, 50 mM NaCl (pH 7.5) was mixed with purified chimeras 206 and 264 in 50 mM Hepes (pH 7.5) in a ratio of 180:1 (equimolar amounts of PA_83_ and VWA domains). Following incubation for 20 min at room temprature, an aliquot from each of the samples was removed and stored at −20 °C pending analysis. The remainder of the samples was transferred to an ultracentrifuge tube and underlayed with a 30% (w/w) sucrose cushion in 50 mM Hepes (pH 7.5). Complexes of chimeras decorated with PA_83_ were pelleted by centrifugation at ∼200,000*g* for 45 min. The complexes were resuspended in 50 mM Hepes, mixed with SDS loading buffer, and heated at 95 °C for 10 min. Aliquots were electrophoresed through a 4%–12% Bis-Tris polyacrylamide gel, in parallel with the aliquots taken before pelleting. The gels were stained with Simply Blue (Invitrogen). The amount of protein in each band was determined by densitometric analysis using FluorChem SP (Alpha Innotech).

### Cell intoxication assay with CHO-K1 cells.

Cell intoxication studies were performed in CHO-K1 cells as described previously [10]. Briefly, 5 × 10^3^ cells in 100 μl of Hams-F12 nutrient mixture (Gibco BRL) supplemented with 10% fetal bovine serum were plated into wells of a 96-well microtitre plate a day prior to the assay. Varying amounts of FHV-VWA_ANTXR2_ VLP or soluble ANTXR2 [8] were preincubated for 20 min in 100 μl of medium containing PA and LF_N_-DTA at a molar concentration of 10^−8^ and 10^−10^, respectively. The mixture was added to the cells, which were incubated at 37 °C for approximately 40 h. The medium was then replaced with 50 μl of Celltiter-glo reagent (Promega) diluted 1:1 with PBS. Luciferase activity as a measure of cell viability was determined with a luminometer (TopCount NXT, Perkin Elmer). Non-linear regression analysis was used to calculate IC_50_ values (Prism, GraphPad Software).

### Marophage-based toxin neutralization assay.

RAW264.7 cells (5 × 10^4^) were plated in each well of a white 96-well tissue culture plate (Corning Costar) with 100 μl of Dulbecco's Modified Eagle Medium (Gibco) supplemented with 10% standard fetal bovine serum the day before the assay. Varying amounts of FHV-VWA_ANTXR2_ VLP were preincubated for 20 min in 400 μl of medium containing PA and LF at a molar concentration of 10^−8^ and 10^−9^, respectively. The mixture (100 μl) was added to the cells in triplicate and incubated at 37 °C for approximately 5 h. Cell viability was determined as described for CHO-K1 cells.

###  LeTx challenge with FHV-VWA_ANTRX2_ VLPs.

LeTx challenge experiments were performed in cannulated, male Fisher 344 rats (180–200 g, Harlan) according to protocols approved by the Scripps Institutional Animal Care and Use Committee. LeTx for each rat was prepared by mixing 20 μg of PA and 8 μg of LF (List Biological Laboratories). All rats were anesthetized with isofluorane and inoculated through a jugular vein cannula with 500 μl of LeTx (control), or LeTx premixed with FHV-VWA_ANTXR2_ VLPs or sANTXR2 (test). Additional rat control groups were injected with either PBS or wt FHV. Rats recovered from anesthesia within 5 min after dosing and were monitored for symptoms of intoxication (agitation, respiratory distress, hypoxia) and death as determined by cessation of respiration.

### Statistical analysis.

Data are presented as mean ± SD. Significance was reported using the Student's unpaired *T*-test (Prism, GraphPad Software). *p*-Values <0.05 were considered statistically significant. Data were also analyzed using 2-way ANOVA followed by Tukey's post-comparison test to confirm significance.

### Preparation of FHV-VWA_ANTRX2_ chimera 264-PA_83_ complexes.

Purified chimera 264 VLPs in 50 mM Hepes (pH 7.5) were mixed with a 4-fold molar excess of recombinant PA_83_ (List Biological Laboratories) in 5 mM Hepes, 50 mM NaCl (pH 7.5), and incubated at room temperature for 20 min with mild agitation. The sample was then transferred to an ultracentrifuge tube and underlayed with a 30% (w/w) sucrose cushion in 50 mM Hepes (pH 7.5). Complexes of chimera 264 decorated with PA_83_ were pelleted by centrifugation at 197,568*g* at 11 °C in an SW41Ti rotor for 1.5 h or an SW 55 Ti rotor for 45 min. The complexes were resuspended in 50 mM Hepes and analyzed by electrophoresis to confirm the presence of chimera 264 and PA_83_ proteins.

### Rat immunization and LeTx challenge.

Immunization studies were performed in male Harlan Sprague Dawley rats (180–200g, Harlan) according to protocols approved by the Scripps Institutional Animal Care and Use Committee. For double dose immunization, rats were injected subcutaneously with 200 μl containing either 2.5 μg of PA_83_ (List Biological Laboratories), 5.4 μg of FHV- VWA_ANTXR2_-264-PA_83_ (molar equivalent of 2.5 μg PA_83_), or 2.9 μg FHV-VWA_ANTXR2_-264 (molar equivalent of particles complexed with PA_83_) all prepared in PBS. For single dose immunization, rats were injected subcutaneously with 200 μl containing either 5 μg of PA_83_, 10.8 μg of FHV-VWA_ANTXR2_-264-PA_83_ (molar equivalent of 5 μg PA_83_), or 5.8 μg of FHV-VWA_ANTXR2_-264 (molar equivalent of particles complexed with PA_83_) all in PBS. Rats were anesthetized with isofluorane before all procedures. Serum was prepared from blood (200 μl per rat) collected from the retro-orbital plexus before immunization, 3 wk post primary immunization, and 4 wk post secondary immunization (study 1 only). LeTx challenge experiments were performed 13 wk post initial immunization for the double dose immunization study and 4 wk post immunization for the single dose immunization study. LeTx for each rat was prepared by mixing 40 μg of PA and 8 μg of LF (List Biological Laboratories) in PBS. Rats were anesthetized with isofluorane and inoculated with 500 μl of LeTx or PBS (control) by intravenous tail vein injection. Rats recovered from anesthesia within 5 min after dosing and were monitored for symptoms of intoxication and death as described above.

### Antibody detection.

Serum samples were tested for antibody response to PA, ANTXR2, and FHV by ELISA assays. Briefly, microtitre Immulon 2HB 96-well plates (Dynex Technologies) were coated with 100 μl of 10 μg/ml PA_83_, sANTXR2, or FHV in coating buffer (0.1 M NaHCO3 [pH 8.5]), blocked with 3% non-fat milk in TBS, and incubated with serum samples diluted 1:100 and 1:1,000 in 1% non-fat milk in TBS (pH 7.0) with 0.05% Tween 20 for 1 h at room temperature. After washing, wells were incubated with biotin-SP-conjugated donkey anti-rat IgG (Jackson ImmunoResearch) diluted 1:20,000 for 1 h at room temperature. Plates were washed and incubated for 45 min with streptavidin-alkaline phosphatase conjugate (GE Healthcare Bio-Sciences) diluted 1:5,000 in TBS. After washing, plates were incubated with p-nitriphenyl phosphate (Sigma-Aldrich) at 37 °C for 20 min. 2 N NaOH was added to stop the reaction and the signal was quantified using an ELISA reader (Molecular Devices) at 405 nm.

## Supporting Information

Figure S1 3-D Surface-Shaded Reconstructions of (A) FHV-VWA_ANTXR2_ Chimera 206, (B) FHV-VWA_ANTXR2_ Chimera 264, and (C) wt FHV Note the protruding surface densities on the chimeric VLPs due to the addition of the VWA domain of ANTXR2. Bar = 100 Å.
(304 KB TIF)

Figure S2 Pseudoatomic Models of FHV-VWA_ANTXR2_ Chimeras X-ray coordinates of FHV capsid protein (green) and ANTXR2 VWA domain (yellow) were docked into the cryoEM density (grey) of chimera 206 (A) and chimera 264 (B). Shown are octants of the density maps to illustrate the good fit of the high resolution X-ray structures into the cryoEM maps. Density within the interior of the capsid shell is ascribed to the encapsidated RNA.
(794 KB TIF)

Figure S3 *In Silico* Model of PA_83_ Bound to the Surface of FHV-VWA_ANTXR2_ Chimeras A maximum number of PA_83_ molecules (purple) was modeled onto the surface of chimera 206 (A) and chimera 264 (B), as limited by steric hindrance. Modeling was based on the known X-ray crystal structures of FHV [17] and ANTXR2-VWA/PA_63_ complex [9]. The FHV capsid protein and the VWA domain of ANTXR2 are in green and yellow, respectively. Panels show cross-sections of the chimeric particles, including the cryoEM density map (grey).
(959 KB TIF)

Figure S4 Quantification of PA_83_ Bound to Chimera 206 and Chimera 264 (A) Recombinant PA_83_ was mixed with purified chimeras 206 and 264 in a ratio of 180:1 (equimolar amounts of PA_83_ and VWA) and an aliquot from each sample was removed and stored. The remainder of the sample was centrifuged through a sucrose cushion to remove unbound PA_83_ and the pellet was resuspended in electrophoresis buffer. Aliquots were electrophoresed through a 4%–12% Bis-Tris polyacrylamide gel in parallel with aliquots taken before pelleting. The gels were stained with Simply Blue (Invitrogen). Lane 1, molecular weight markers; lane 2, PA_83_ and chimera 206 before pelleting; lane 3, PA_83_ and chimera 206 after pelleting; lane 4, PA_83_ and chimera 264 before pelleting; lane 5, PA_83_ and chimera 264 after pelleting. (B) Quantitative representation of PA_83_ associated with chimeric particles after pelleting. Protein representing PA_83_ and chimeras 206 and 264 shown in (A) was quantified by densitometric analysis using FluorChem SP (Alpha Innotech) and the number of PA molecules bound to the particles calculated. The numbers were normalized to the amount of protein in the respective samples before pelleting.
(963 KB TIF)

### Accession Numbers

The GenBank (http://www.ncbi.nlm.nih.gov/Genbank/) accession numbers for the proteins discussed in this paper are Flock House virus coat protein (NC004144) and human capillary morphogenesis protein 2 (CMG2) (AY23345).

Protein Data Bank (http://www.rcsb.org/pdb/) accession numbers are for the VWA domain of CMG2 (1SHT) and B. anthracis protective antigen PA_63_ complexed with CMG2 (1T6B).

The Virus Particle Explorer (http://viperdb.scripps.edu/) was used for Flock House virus particle coordinates.

## Abbreviations

AVA: anthrax-vaccine adsorbed
EF: edema factor
FHV: Flock House virus
LeTx: lethal toxin
LF: lethal factor
MLD: minimum lethal dose
PA: protective antigen
VLP: virus-like particle
VWA: von Willebrand A
wt: wild-type

## Note Added in Proof

References [9] and [10] are cited out of order and appear later in the article than where numerical order would put them.
